# Genetic testing and diagnosis of inherited retinal diseases

**DOI:** 10.1186/s13023-021-02145-0

**Published:** 2021-12-14

**Authors:** Byron L. Lam, Bart P. Leroy, Graeme Black, Tuyen Ong, Dan Yoon, Karmen Trzupek

**Affiliations:** 1 Bascom Palmer Eye Institute, University of Miami Miller School of Medicine, 900 NW 17th Street, Miami, FL 33156 USA; 2 Department of Ophthalmology, Ghent University and Ghent University Hospital, Ghent, Belgium; 3 Ophthalmic Genetics and Visual Electrophysiology, Division of Ophthalmology, Children’s Hospital of Philadelphia, Philadelphia, PA USA; 4 UK Inherited Retinal Disease Consortium, Manchester, UK; 5 Genomics England Clinical Interpretation Partnership, Manchester, UK; 6 Manchester Centre for Genomic Medicine, St. Mary’s Hospital, Manchester, UK; 7 Ring Therapeutics, Cambridge, MA USA; 8 Biogen, Cambridge, MA USA; 9 Ocular and Rare Disease Genetics Services, InformedDNA, St Petersburg, FL USA

**Keywords:** Inherited retinal disease, Ophthalmology, Molecular diagnosis, Genetic testing, Genetic counseling, Case studies, Next-generation sequencing

## Abstract

Inherited retinal diseases (IRDs) are a diverse group of degenerative diseases of the retina that can lead to significant reduction in vision and blindness. Because of the considerable phenotypic overlap among IRDs, genetic testing is a critical step in obtaining a definitive diagnosis for affected individuals and enabling access to emerging gene therapy–based treatments and ongoing clinical studies. While advances in molecular diagnostic technologies have significantly improved the understanding of IRDs and identification of disease-causing variants, training in genetic diagnostics among ophthalmologists is limited. In this review, we will provide ophthalmologists with an overview of genetic testing for IRDs, including the types of available testing, variant interpretation, and genetic counseling. Additionally, we will discuss the clinical applications of genetic testing in the molecular diagnosis of IRDs through case studies.

## Introduction

Inherited retinal diseases (IRDs) are a heterogenous group of visually debilitating diseases caused by pathogenic variation in proteins critical to retinal function. The majority of IRDs are characterized by retinal degeneration, which can lead to significant vision impairment and blindness [1–4]. Collectively, IRDs are estimated to affect more than 2 million people worldwide [5, 6]. Because of the phenotypic overlap of several IRDs, establishing a definitive clinical diagnosis may be difficult [7]. Therefore, molecular genetic testing has become an important strategy to complement clinical findings and confirm or clarify a diagnosis.

With an increased understanding of the human genome and the wide scope of genetic variants identified to be associated with IRDs (> 250 causative genes) [8], emerging genetic testing technologies such as next-generation sequencing are allowing clinicians to better diagnose IRDs [9–11]. The advent of these sophisticated testing technologies for genetic disorders has highlighted the need for broader awareness of human genetics and its relevance to personalized medicine in IRDs.

The American Academy of Ophthalmology Task Force on Genetic Testing and the European Reference Network for Rare Eye Diseases recommend genetic testing for all individuals with presumed or suspected IRDs for which a causative gene or genes have been identified [12, 13]. However, most clinical ophthalmologists are unfamiliar with genomic diagnostics. In a retrospective analysis of genetic testing utilization for individuals with IRDs within a large, university-based health system conducted from 2008 to 2018, providers felt that genetic testing was useful in IRD management [14]. However, the analysis found that genetic testing was infrequently utilized, with only 1.5% of 207 individuals with IRDs undergoing genetic testing ordered from an ophthalmologist’s office [14].

Historically, genetic testing has been ordered and interpreted by IRD specialists and ocular genetic counselors at large academic research centers [12]. However, this structure does not adequately meet patient demand for 3 major reasons. First, there are not enough IRD specialists and ocular genetic counselors at academic medical centers to meet patient demand for genetic testing. While there are currently close to 5000 certified genetic counselors in the United States, < 1% of them have expertise in ophthalmology [15]. Second, many individuals with IRDs are geographically isolated from those centers and unable or unwilling to travel. It is important for affected individuals to be evaluated by a retina specialist who is familiar with IRDs and has the expertise to make a provisional clinical diagnosis. Community-based retina specialists have successfully incorporated genetics into their practice by ordering testing themselves; however, the importance of both pre- and post-test genetic counseling should be recognized. Particularly in the United States, telemedicine-based genetic counseling services are becoming more widely available to support geographically or economically disadvantaged individuals in accessing specialists in ocular genetics [16]. This allows community-based retina specialists to partner with telephone-based genetic counselors to help disclose results to individuals and manage conversations regarding complex results and risks to family members. Lastly, the global use of genetic testing is limited because of the challenge for the budgets and structure of regional healthcare systems to cover the cost of genetic testing, particularly in countries with low resources [13].

Early and accurate diagnosis is necessary for individuals with IRDs to enable patient decision-making, to identify suitable clinical studies or treatment opportunities, and to improve patient outcomes. The objective of this review is to provide a general understanding of genetic testing and its clinical applications for IRDs through case studies.

## Genetic testing

Genetic testing is the analysis of an individual’s DNA to detect genetic changes or variants that could lead to disease [17]. With the application of improved molecular testing technology, the likelihood of identifying a causative variant in individuals with IRDs has increased [10, 11, 18–20]. Identifying the disease-causing variant can not only illuminate or confirm a diagnosis but can also improve medical management by informing prognosis, reducing the need for additional electrophysiologic testing, clarifying guidance in ocular surveillance, and advising appropriate changes in therapies and/or supplementation [19]. Genetic testing also allows for accurate identification of inheritance pattern, thereby improving genetic counseling for affected individuals and their families [19]. Additionally, with an approved retinal gene therapy for biallelic *RPE65* mutation-associated retinal dystrophy, Luxturna^®^ (voretigene neparvovec-rzyl) [21, 22], and several ocular gene therapy clinical studies in progress [23–25], confirming a molecular diagnosis through genetic testing may help individuals access the latest treatment options or qualify for study participation [2, 7].

When a person has been clinically diagnosed with a presumed IRD, diagnostic genetic testing is indicated. At this time, genetic testing should not be used to rule out an IRD, since a negative result from large panel genetic tests could reflect the limits of the panel’s design and not the comprehensive spectrum of potential genetic candidates [12]. In many cases, diagnostic testing is recommended on the basis of physical symptoms or findings from clinical examination. If an individual is asymptomatic but a disease-causing molecular variant has previously been confirmed in a family member, genetic testing can be used for predictive testing or carrier testing [26]. Predictive testing is used to detect genetic mutations in individuals before they present with symptoms to assess future risk of disease [12, 26]. When no therapeutic intervention exists, predictive testing should be approached with caution, particularly in children. For many IRDs, reduced penetrance or a wide degree of variability in clinical symptoms is known to occur and can complicate the interpretation of results for asymptomatic individuals. The American College of Medical Genetics and Genomics and the American Academy of Pediatrics explicitly discourage the routine testing of at-risk minors for adult-onset conditions with no available therapy or change in management [27]. Carrier testing is used to identify individuals who carry a single pathogenic variant in a recessive or X-linked disease gene that, when present with another pathogenic variant, can cause genetic disease. Carrier testing may be performed on individuals who do not present with symptoms but may be at risk for passing a genetic disease on to their children [26].

Genetic tests can assess single genes or, more commonly, panels of genes associated with a group of genetic diseases [2]. Previously, genetic testing was primarily performed on a single-gene basis, where the small number of genes tested were those determined to be most likely associated with disease on the basis of clinical assessment [7, 19, 28]. This often produced a low diagnostic yield [7]. While traditional single-gene sequencing may be sufficient for diagnosing IRDs with mostly only one disease-associated gene identified, such as congenital anirdia (*PAX6* gene), systematic testing of single genes may be inefficient for diagnosing more complex IRDs that have a high degree of genetic heterogeneity, such as retinitis pigmentosa (> 100 identified causative genes) [2, 7, 8, 12, 19, 29]. With the introduction of next-generation sequencing, testing multiple genes in a single assay has become possible [9]. Next-generation sequencing sequences millions of DNA fragments in parallel and matches them to a reference genome using bioinformatics to detect variants [30, 31]. Next-generation sequencing can be used to completely sequence an individual’s DNA (whole genome sequencing) or to sequence just the protein-coding regions (whole exome sequencing) [30–32]. In the clinical assessment of IRDs, next-generation sequencing can also be used to constrain sequencing to just the coding regions of genes known to cause retinal diseases using a targeted multigene panel [30, 31].

In the processing of genetic test results, identified variants are analyzed to determine their potential association with an IRD phenotype. Not all identified variants cause disease [33]. Genetic variant interpretation, as defined by the American College of Medical Genetics, is classified using a 5-class system (Table 1) [33, 34]. Variants may be classified as benign on the basis of several criteria, including well-established functional data showing no damaging effect of the variant on protein function or splicing, lack of segregation in affected members of a family, and variant frequency in the general population [34]. Variants classified as “likely benign” are those with an estimated > 90% certainty of being benign [34]. Pathogenic variants are disease-causing mutations [34]. Variants classified as “likely pathogenic” are those with an estimated > 90% certainty of causing disease [34]. If a variant does not fulfill benign or pathogenic criteria, or if the evidence for benign and pathogenic are contradictory, the variant defaults to being classified as uncertain significance [34]. Variants of uncertain significance should be reported with caution, and additional testing or iterative clinical investigations may be required to assess the effects of these variants [34]. Processing of genetic findings should always be done in the context of the clinical phenotype [35].

**Table 1:** Summary of reported variant classification and interpretation [34]

Result	Variant interpretation
Benign	Clearly not disease-causing
Likely benign	Unlikely to be disease-causing
Uncertain significance	Evidence is insufficient to support or reject pathogenicity, and additional data are needed
Likely pathogenic	Likely to be disease-causing
Pathogenic	Clearly disease-causing

## Case study 1

**Fig. 1:**
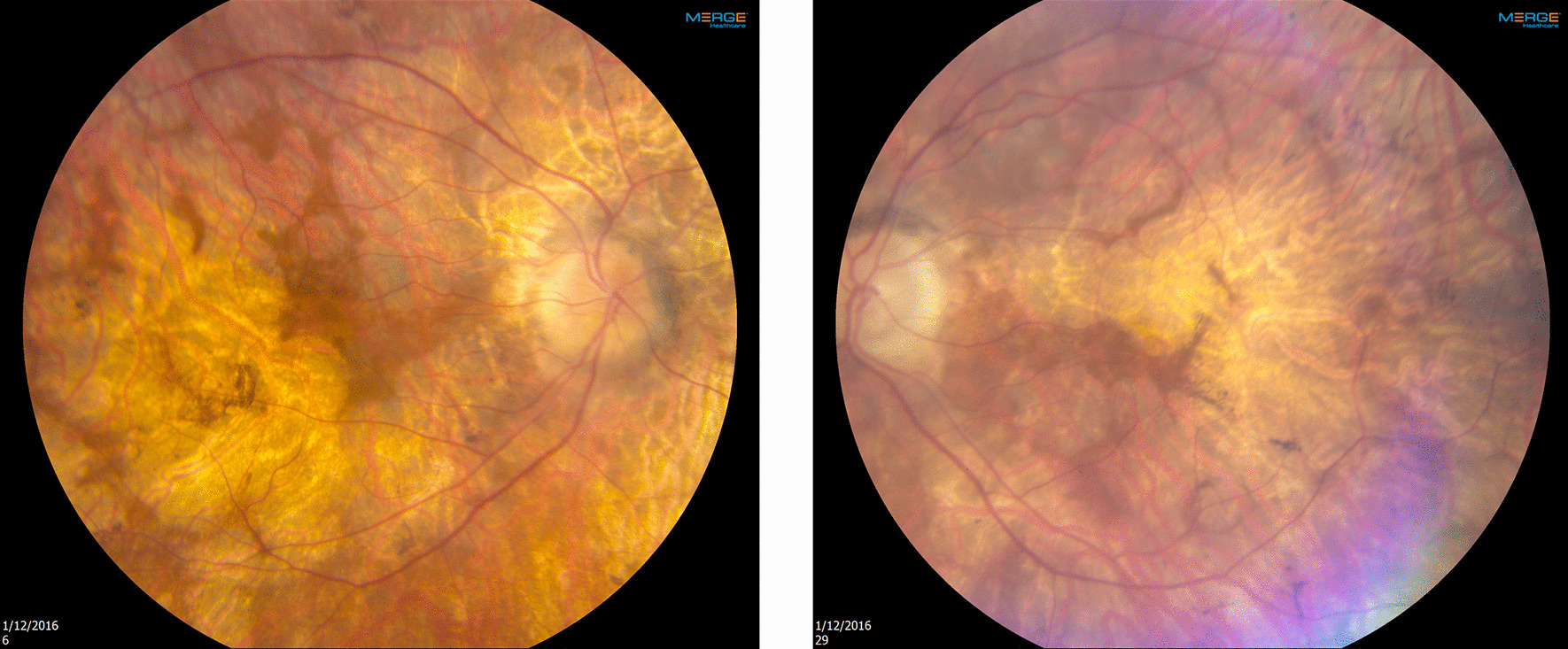
Fundoscopic images from a 60-year-old healthy male with a clinical diagnosis of choroideremia. The genetic testing with next-generation sequencing panel showed the individual was negative for pathogenic mutations of the *CHM* gene and positive for 2 novel heterozygous mutations of the *C2orf71* gene

A 60-year-old healthy male with progressive night visual impairment and peripheral visual loss for many years and a diagnosis of choroideremia was referred for evaluation as a participant for a choroideremia natural history study. Family history was negative. Best-corrected visual acuity was 20/50 in each eye. Funduscopic findings showed areas of well-demarcated chorioretinal atrophy, with small islands of retained functional area, in the macula of each eye (Fig. 1). The working diagnosis was choroideremia; the genetic testing with next-generation sequencing panel showed the individual was negative for pathogenic mutations of the *CHM* gene and positive for 2 novel heterozygous mutations of the *C2orf71* gene, namely, p.Arg1202Ter (nonsense mutation) and p.Ser134ArgfsTer47 (frameshift mutation). Mutations of the *C2orf71* gene cause autosomal recessive retinitis pigmentosa; the 2 novel *C2orf71* genetic variants found were considered likely positive. The individual was counseled regarding the hereditary pattern and the prognosis of his *C2orf71*-associated IRD and will be considered a candidate for future *C2orf71* clinical trials.

Pedigree analyses are an important complement to molecular assessment of IRDs [36, 37]. These analyses can support the results of clinical phenotyping, inform reproductive risks, and help guide genetic testing [38]. However, it is important to consider that some IRDs can be difficult to predict on the basis of family history alone. For example, approximately 50% of individuals with retinitis pigmentosa have no known family history of the disease [31, 39], and although most will have autosomal recessive inheritance, up to 10% may have X-linked inheritance and approximately 24% will have dominant inheritance with no known affected family members as the result of reduced penetrance or a de novo genetic variant [39]. Therefore, different patterns of inheritance and associated causative genes should not be ruled out without molecular confirmation.

## Case study 2

**Fig. 2:**
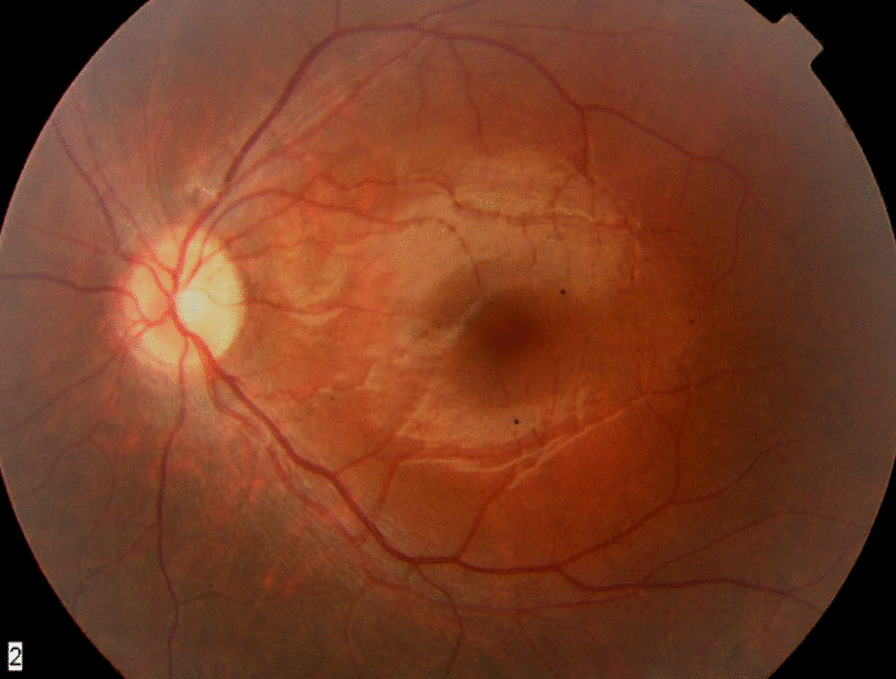
Funduscopic images from 7-year-old boy with a clinical diagnosis of *RPGR*-mutated X-linked retinitis pigmentosa

A 7-year-old male suspected of difficulty with night vision was found to have 20/20 vision bilaterally with normal appearing maculas (Fig. 2). He reported no vision problems and has 2 brothers who were asymptomatic. The individual’s next-generation sequencing panel genetic testing showed a hemizygous pathogenic mutation in the ORF15 region of the *RPGR* gene on the X chromosome (c.2270_2271delAG; p.Glu757GlyfsTer12; this means a deletion of nucleotides A and G at positions 2270 and 2271 in the exon leading to a complete change in amino acid sequence starting at the change of position 757 from glutamic acid to glycine until an early termination after 12 changed amino acids; see https://varnomen.hgvs.org/recommendations/protein/variant/frameshift/ for description of mutation nomenclature). Subsequent testing of his siblings showed a similar genotype. The genetic testing revealed that all 3 siblings actually had X-linked retinitis pigmentosa due to homozygous mutations in the *RPGR* gene. After 16 years, at the age of 23 years, the individual had a vision of 20/40 bilaterally with significant constricted visual fields and was enrolled into a clinical trial for *RPGR* gene therapy.

## Genetic counseling

Prior to coordination of genetic testing, a provider familiar with the genetics of retinal diseases (often an IRD specialist or an ocular genetic counselor) should outline the benefits, limitations, and potential implications of genetic testing with affected individuals and their caregivers [37, 40]. For example, individuals should be made aware that targeted genetic testing may miss some differential diagnoses because of phenotypic overlap among various IRDs [7, 41]. Conversely, it should also be considered that broader testing strategies may increase the likelihood of unexpected or unclear results and that conditions that seem isolated may actually be syndromic [7]. It is important for individuals to understand that receiving genetic testing does not guarantee that they will receive a molecular diagnosis for their IRD, and a positive genetic test result will not necessarily qualify them for a clinical trial or therapy [37]. As not all of the genes and variants associated with IRDs have been identified, testing may not detect the disease-causing variant for all individuals [37]. After the pretest consultation with the counselor, individuals must indicate informed consent to proceed with genetic testing [36].

## Case study 3

A 59-year-old female was referred for genetic counseling of her IRD. She noticed significant night blindness at age 17 and was diagnosed with retinitis pigmentosa at age 28. Further, at the age of 28, she was diagnosed with polycystic kidney disease and underwent a unilateral kidney transplant 2 days later. Genetic testing with a limited panel of 31 genes implicated in early onset retinal disease was ordered before referral for genetic counseling and indicated that she was positive for a heterozygous pathogenic variant in the *RPE65* gene (c.11 + 5G > A). The ordering provider incorrectly interpreted the positive test result as diagnostic of *RPE65*-related IRD, changed the diagnosis to Leber congenital amaurosis, and advised her that she would likely qualify for treatment with Luxturna.

During the genetic counseling appointment, the individual disclosed that she had 2 brothers who died of kidney disease in late childhood. Subsequent additional genetic testing revealed a complete deletion of the *NPHP1* gene (interpreted as pathogenic) in the homozygous state. The individual's retinal disease, kidney disease, and family history are consistent with a syndromic form of retinal dystrophy due to the *NPHP1* gene. The presence of a pathogenic variant in the *RPE65* gene indicates that she is additionally a carrier of recessively inherited *RPE65*-related retinal dystrophy, a state that does not qualify her for Luxturna gene therapy.

Post-test genetic counseling is also important for interpreting and discussing genetic test results in light of patient history [19, 37]. Accurate interpretation of genetic test results is very important and helps health care providers advise individuals about their prognosis and provide more clarity about how an eye disease is inherited [12]. As the number of genes commercially available for sequencing increases and the use of genetic sequencing processes becomes more widespread, accurate interpretation of the results from these tests is critical [34]. On the basis of findings from the genetic test, counselors will also advise on the implications of results for at-risk family members, offer appropriate screening or preventative strategies, and educate on other potential health or lifestyle effects [37, 40]. As previously discussed, results from genetic testing can help providers direct individuals to available targeted therapies and/or establish eligibility for participation in applicable ongoing clinical studies [19].

## Case study 4

A female with progressive impairment of night vision from late childhood, legal blindness by age 16, and a diagnosis of severe nonsyndromic retinitis pigmentosa was referred for evaluation. Genetic testing showed that she was positive for 2 heterozygous mutations of the *BBS1* gene, p.Met390Arg (pathogenic) and p.Glu224Lys (likely pathogenic), resulting in a diagnosis of Bardet-Biedl syndrome. This condition is associated with multiple risks of other health conditions, requiring significant medical follow-up. The genetic counselor provided medical management guidelines established for Bardet–Biedl syndrome to the patient, the referring retina specialist, and the primary care physician. These include regular ophthalmic evaluations, body mass index calculations, diabetes testing, and lipid profiling.

Barriers to genetic testing remain a challenge for individuals with IRDs and health care professionals. One significant challenge for the widespread implementation of genetic testing is the varied payment/reimbursement mechanisms across countries [42, 43]. In some countries, there is limited coverage of genetic testing costs [1, 42]. Insurance companies and other funding agencies may be reluctant to pay for genetic testing unless there is clear evidence that the results will affect medical management [31]. Another significant barrier is limited patient access to expert providers and genetic testing services, especially in underserved and remote areas [44]. Without guidance from an IRD or genetics expert, ordering and interpretation of genetic tests can be complex. Because of the functional limitations associated with having an IRD, affected individuals may need a caregiver to facilitate access to genetic testing services and appointments with genetic counselors or IRD specialists. This could result in loss of workdays or working time for both the affected individual and caregiver [45]. Another challenge is ophthalmologists’ generally limited knowledge of genetic services, genetic conditions, and patient risk factors [44]. This lack of knowledge can lead to insufficient referrals from ophthalmologists to IRD experts and appropriate genetic services. Inadequate completion of family history analysis can also impede genetic testing efforts [44]. While a thorough family history can aid decision-making and inform genetic testing approaches, incomplete assessment may delay testing or lessen the likelihood of identifying a causative mutation. Finally, difficulties consolidating genetic data into public databases can also be a hurdle for genetic testing [1, 46, 47]. While large databases are commonly used by diagnostic laboratories, entries may not be complete or up to date [46, 47]. Variant data may be stored in local or specialized databases and not collated in a multicenter database that can be easily accessed or shared [46, 47].

Despite the complexity of genetic tests and their practical limitations, it is necessary to overcome these barriers to ensure that the best medical care is provided for individuals affected by IRDs. Some potential approaches to overcome these barriers and improve the patient’s experience include expanding education of health care professionals about IRDs and genetic testing options, creating tools to connect affected individuals with experts in IRDs [48], and improving data sharing via strategies to support streamlined and secure distribution of genomic variant data from clinical laboratories [1]. A valuable service that has transformed health care in recent years is telemedicine [16]. Telemedicine can facilitate medical education, e-health patient monitoring, and patient consultation through which ocular genetic counselors can provide remote counseling to overcome geographical constraints and expand access to patient care [16, 49].

## Genetic testing recommendations for individuals with IRDS

**Fig. 3:**
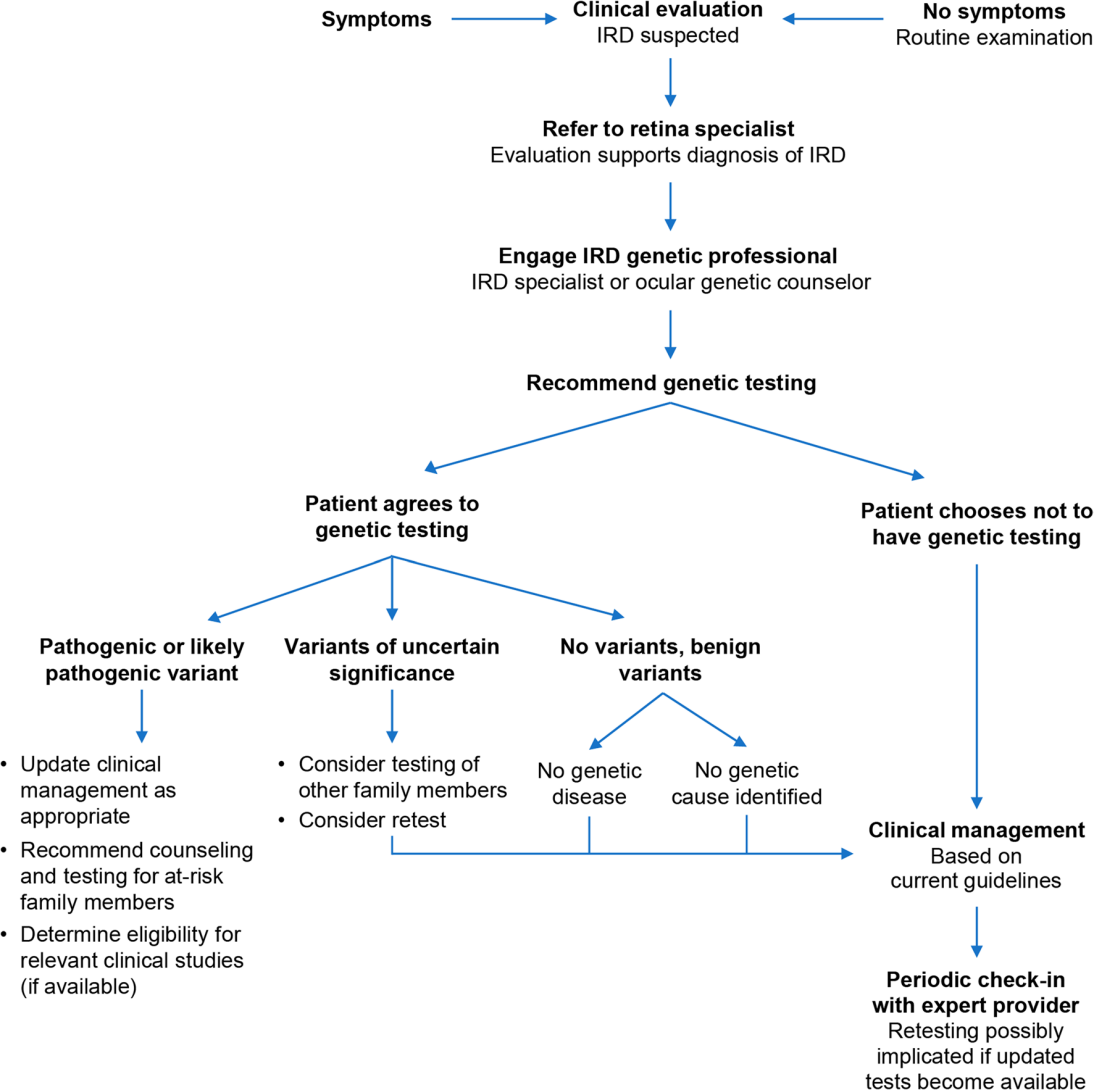
Decision tree for case scenarios. IRD, inherited retinal disease

To enhance genetic disease management, a decision tree algorithm may be useful for easy reference in clinical practice to help determine the indications for molecular diagnosis in individuals with IRDs (Fig. 3). The American Academy of Ophthalmology recommendations for the genetic testing of IRDs state that whenever clinical findings suggest the possibility for an IRD for which a causative gene or genes have been identified, the treating ophthalmologist should either discuss the value of genetic testing and order an appropriate test (if available) or refer the individual to an IRD specialist or ocular genetic counselor with expertise in the selection and interpretation of molecular tests [12].

Retina specialists or ocular genetic counselors will review the individual’s clinical, ocular, and family history [37]. In some cases, additional clinical assessments may be advised. If evaluation supports the diagnosis of an IRD and genetic testing is considered appropriate after pretest counseling, a genetic test will be ordered. If a pathogenic variant is identified, the specialist or counselor will assess the result in the context of the individual’s family and clinical history to confirm diagnosis and will provide actionable recommendations including medical management strategies. Ideally, individuals with IRDs should be assessed annually for associated, treatable findings such as cataracts and cystoid macular edema. Individuals with IRDs are also advised to return for follow-up expeditiously if there is an acute change in vision.

If testing reveals a variant of uncertain significance, additional clinical data may be collected or family members may be tested; however, this does not always help to elucidate the effect of the variant [34]. Variants of uncertain significance are almost always found with next-generation sequencing panel testing or whole exome sequencing, with or without applied filters. Family member testing can be pursued for parental or segregation analysis if additional family members are available to test. The absence of a genetic variant in both parents of a child can prove de novo inheritance, which provides strong evidence of pathogenicity. In dominant and X-linked conditions, if unequivocal co-segregation of a clinical phenotype with a variant is established in at least 3 generations, then a variant of uncertain significance is highly likely to be pathogenic and reclassification of a Class 3 (variant of uncertain significance) to Class 4 or 5 (pathogenic or likely pathogenic) is warranted. Further, identification of gene-specific phenotype features (e.g., flat electro-oculography in a patient with vitelliform dystrophy and a BEST1 variant) can also support variant interpretation. If phenotypic clinical findings suggest more than one specific genotype, the variant of unknown significance can sometimes be reassessed on the basis of the effect on gene and protein structure and function to determine potential pathogenicity. Direct assays may be useful in cases in which variants are thought to alter RNA splicing; however, functional assessments may be time-consuming and expensive. Structural assessments such as in silico analysis may be valuable; however, they should generally only be used to support other lines of evidence.

If test results are negative for pathogenic variants, this could either indicate that the individual does not have a genetic cause of their disease [50] or, more often the case, that the molecular cause of the disease has not been identified. It is possible that individuals receiving a negative genetic test result may harbor variants in currently unknown IRD genes or in regions of known genes not currently identified using current testing methodologies. Genetic reevaluation may be valuable and, because new pathogenic genetic variants are continually being identified, may be considered every 2–5 years. Retesting may be dependent on the mode of genetic analysis initially utilized. Reanalysis of existing data (e.g., in cases in which patients have had whole exome or whole genome analysis) may be simpler and cost less than a complete reevaluation. In cases in which a new genetic test is conducted, it is important to assess whether it will be substantially different than previous analyses, as diagnostic tests may not be updated regularly. In all cases, coordination between the primary provider and retina specialist, and/or genetic counselor, is critical to facilitate genetic testing and guide appropriate care for individuals with IRDs. Table 2 provides some useful websites regarding basic management of genetic disorders, as well as genetic counseling, genetic testing, and available supportive services.

**Table 2:** Useful websites

Website	Description
GeneReviews https://www.ncbi.nlm.nih.gov/books/NBK1116/	Provides detailed information on clinical scenarios and specific single-gene disorders, authored by experts. These articles also contain recommendations on basic management, genetic counseling, and genetic testing
VisionServe Alliance https://visionservealliance.org/vision-loss-resources/lost-your-vision/	Provides a listing of local support services for those with vision impairment or blindness

## Conclusions

Inherited retinal diseases present challenges in molecular diagnostics because of their high genetic heterogeneity, overlapping clinical presentations, and variability in inheritance patterns. Currently, ophthalmologists who come across individuals experiencing symptoms consistent with an IRD may benefit from the expertise of an ocular genetic counselor or IRD specialist. With the complexity of IRDs, collaboration with specialists may allow for improved decision-making regarding the most appropriate genetic test panel, pre- and post-test counseling, and accurate interpretation of the test findings in the context of the clinical diagnosis. While adequate genetic testing resources and specialty providers may be unavailable in many regions, improved understanding of the genetics within ophthalmic subspecialties and increased use of telemedicine-based options may help meet the needs of individuals to fully understand their diagnosis.

## Data Availability

Data sharing is not applicable to this article as no data sets were generated or analyzed during the current study.
